# Is a Workable Scheme Still to Be Found?

**Published:** 1921-05-07

**Authors:** 


### THE KING'S FUND AND INCOME TAX.

### Is a Workable Scheme Still to be Found?

Before Lord Cave's Committee was appointed King Edward's Hospital Fund for London requested a Sub-Committee to consider the question of securing exemption of contributions to scheduled charities from assessable income for income-tax purposes, and to examine a proposal that the King's Fund might take the initiative in promoting a combined application by representative charitable funds. This Sub-Committee have reported to the Fund, and suggest that the report should be submitted to Lord Cave's Committee. We regret that space does not allow a detailed account of the report of the Sub-Committee, but we can state that they have considered three schemes, and, having decided that the general principle of income-tax relief might be adopted, suggest that, subject to certain limitations, a hospital should be relieved in respect of income tax paid by subscribers and donors in cases where the hospital has received from the contributor a certificate stating that income tax has been paid on the contribution, and declaring his desire that relief should be granted to the hospital. A form of equalisation amongst hospitals, in order that some should not wax too fat under the new order of things, is also proposed. It is calculated that the maximum liability in respect of London, under a flat rate of 4s. in the £, would be £191,000. It must be said at once that few who possess inside knowledge of hospital accounts and the psychology of subscribers and donors could seriously hope that such a scheme would be worth the trouble of working, despite the possibility of obtaining more of the larger donations. Most of the charitable contributions from income-tax payers come in the form of small sums, perhaps from one to twenty pounds. To apply the scheme with any profit each of this multitude of persons would be asked to sign a certificate; many of them several certificates. The risk of losing many subscribers and potential legators is obvious. Then with regard to equalisation, what hospital is going to keep down renewals and repairs of building and equipment, and salaries of staff, in order to provide a surplus to be drawn upon for the relief of other hospitals? The signatories of the report are Sir Basil Mayhew, Mr. Henry L. Hopkinson, and Mr. John G. Griffiths. Sir Walter Trower dissents, because the suggestion is inconsistent with the report of the Royal Commission on Income Tax, of which he was a member, but we understand he will bow to a decision made by Lord Cave's Committee. Stevenson said: "All my old opinions were only stages on the way to the one I now hold, as itself is only a stage on the way to something else." Certainly in this matter of hospitals we need flexible minds, and we also require full knowledge of detail.

